# Gait Speed as a Predictor for Diabetes Incidence in People with or at Risk of Knee Osteoarthritis: A Longitudinal Analysis from the Osteoarthritis Initiative

**DOI:** 10.3390/ijerph18094414

**Published:** 2021-04-21

**Authors:** Aqeel M. Alenazi, Bader A. Alqahtani, Vishal Vennu, Mohammed M. Alshehri, Ahmad D. Alanazi, Saud M. Alrawaili, Kamlesh Khunti, Neil A. Segal, Saad M. Bindawas

**Affiliations:** 1 Department of Health and Rehabilitation Sciences, College of Applied Medical Sciences, Prince Sattam Bin Abdulaziz University, AlKharj 11942, Saudi Arabia; Ba.alqahtani@psau.edu.sa (B.A.A.); s.alrawaili@psau.edu.sa (S.M.A.); 2 Department of Rehabilitation Sciences, College of Applied Medical Sciences, King Saud University, Riyadh 11433, Saudi Arabia; vvnnu@ksu.edu.sa (V.V.); sbindawas@ksu.edu.sa (S.M.B.); 3 Department of Physical Therapy, College of Applied Medical Sciences, Jazan University, Jizan 45142, Saudi Arabia; phdalshehri@gmail.com; 4 Department of Physical Therapy, College of Applied Medical Sciences, Majmaah University, Al Majmaah 11952, Saudi Arabia; aalanazi@mu.edu.sa; 5 Diabetes Research Centre, University of Leicester, Leicester General Hospital, Gwendolen Road, Leicester LE5 4PW, UK; kk22@leicester.ac.uk; 6 Department of Rehabilitation Medicine, University of Kansas Medical Center, 3901 Rainbow Blvd, Kansas City, KS 66160, USA; nsegal@kumc.edu

**Keywords:** diabetes incidence, knee pain, walking speed

## Abstract

Background: This study examined the association between baseline gait speed with incident diabetes mellitus (DM) among people with or at elevated risk for knee OA. Materials and Methods: Participants from the Osteoarthritis Initiative, aged 45 to 79 years, where included. Participants with or at risk of knee OA from baseline to the 96-month visit were included. Participants with self-reported DM at baseline were excluded. DM incidence was followed over the 4-time points. Gait speed was measured at baseline using a 20-m walk test. Generalized estimating equations with logistic regression were utilized for analyses. Receiver operator characteristic curves and area under the curve were used to determine the cutoff score for baseline speed. Results: Of the 4313 participants included in the analyses (58.7% females), 301 participants had a cumulative incidence of DM of 7.0% during follow-up. Decreased gait speed was a significant predictor of incident DM (RR 0.44, *p* = 0.018). The threshold for baseline gait speed that predicted incident DM was 1.32 m/s with an area under the curve of 0.59 (*p* < 0.001). Conclusions: Baseline gait speed could be an important screening tool for identifying people at risk of incident diabetes, and the determined cutoff value for gait speed should be examined in future research.

## 1. Introduction

Knee osteoarthritis (OA) and diabetes mellitus (DM) are common chronic comorbid conditions [1]. The coexistence of these two conditions can be explained by shared risk factors such as age, obesity, and low physical activity [2,3,4]. In the United States, the prevalence of knee OA is estimated to be 14% in adults aged 25 years and older, and the percentage increases with aging [1,5]. DM affects at least 17% of American adults aged 45 years and older, and 25% of those aged 65 and older [6]. Both knee OA and DM have been associated with reduced gait speed and concomitant functional decline [7,8].

Gait speed is an essential measure of health and a powerful predictor of future disability, morbidity, and mortality [9,10,11]. It has been regarded as “the sixth vital sign” due to its predictive capabilities and excellent psychometric properties [12]. Furthermore, slow gait speed has been associated with frailty, falls, poor quality of life, hospitalization, cognitive decline, and functional dependency [13,14,15]. Individuals with diabetes walk slower (1.3 m/s) than healthy adults (1.4 m/s) [16]. In addition, people with arthritis have a slower gait speed (1.27 m/s) when compared to those without arthritis (1.35 m/s) [17]. However, it is unclear if there is a differential effect of gait speed in the presence of both DM and arthritis. Past research has linked metabolic syndromes to knee OA than hip OA while genetic factors has been linked to hip OA than knee OA [18,19,20]. Individuals with knee OA may tend to reduce their activity and gait speed to manage their symptoms. Previous evidence identified that DM was associated with pain during walk and slower gait speed in people with or at risk of knee OA [21]. Therefore, using gait speed as a predictor for DM has a clinical value to identify and screen adults with knee OA who are at risk.

In a meta-analysis examining the association between OA and DM, the risk of DM in people with OA was 40% higher compared to individuals without OA [22]. In a cross-sectional study, about 47% of the study sample with and without DM had OA, but 6.3% had both DM and OA [23]. Furthermore, the association between DM and OA has not only been linked to specific joints such as knees, hips, or hands [22], but it has been linked to generalized OA (i.e., OA in three or more joints) versus localized OA (i.e., OA in 1–2 joints). Our previous work has found a higher prevalence of DM in people with generalized OA (25%) when compared to the prevalence of DM in people with localized OA (12%) [24]. In a prospective longitudinal study using diagnostic codes, patients with OA had a higher incidence of DM than those without OA [25]. Another study found that self-reported walking difficulty can explain 37–45% of the relationship between OA and DM incidence [26]. However, walking difficulty was measured by self-report (yes or no). Previous research found that walking difficulty using self-reported measures is not equivalent to objective measures for gait speed [27]. Self-reported walking and objective gait speed difficulty may measure different dimensions of the same phenomenon [27]. Furthermore, self-reported difficulty might be affected by several personal and cultural factors affecting the understanding of the “difficulty”. Other limitations in self-reported walking difficulty include the limited precision and recall bias compared to objective measures [28]. Therefore, it is essential to examine this longitudinal association between gait speed and DM incidence in people with OA. Gait speed is a reliable and objective disability indicator, and it is a clinical tool that is sensitive to subtle changes.

Our cross-sectional work revealed that DM was associated with slower gait speed in people with knee OA [29]. However, the cutoff value for gait speed in that study was set at 1.0 m/s based on previous research. Another work has revealed that DM was associated with a 0.064 m/s decrease in gait speed among people with or at risk of knee OA after adjustment of other covariates such as demographics, depression symptoms, OA grade, and knee pain while walking [21]. However, this study was a cross-sectional design that did not account for longitudinal analysis of gait speed and DM and OA. Only one study examined baseline gait speed and DM incidence in Japanese older adults over 4.16 years of follow-up [30]. Although this study found significant association between baseline gait speed and DM incidence, this study has some limitations such as small sample (n = 102), indicating underpower design that cannot be generalized, shorter time of follow-up affecting DM incidence capture, and included only healthy older adults who participated in an exercise program for nine years. Furthermore, this study did not include people with or at risk of knee OA. People with OA and DM are at a greater risk of metabolic syndromes and functional limitations such as higher pain and slower gait speed [21,24,31,32,33,34,35] requiring early intervention. Therefore, it is essential to examine the prognostic utility for gait speed in predicting incident DM, and its associated cutoff value. We are unaware of longitudinal studies investigating the relationship between baseline gait speed and DM incidence in people with or at elevated risk for knee OA.

Given these gaps in the available evidence, in the context of the known relationship between knee OA and DM, this study had three objectives: (1) to examine the association between baseline gait speed as a continuous variable and DM incidence among people with or at risk of knee OA; (2) to determine the cutoff value for gait speed that predicted DM incidence in this population; and (3) to explore whether the specified cutoff value (slower vs. normal gait speed) was a significant predictor of DM incidence. We hypothesized that slower gait speed compared to normal gait speed would be a strong predictor of DM incidence in this cohort.

## 2. Materials and Methods

This study is a longitudinal prospective cohort analysis from the Osteoarthritis Initiative (OAI) from baseline to 96-month follow-up (https://data-archive.nimh.nih.gov/oai/) (accessed on 21 May 2019) OAI is a longitudinal multisite study in the United States. This study enrolled 4796 participants with or at elevated risk for knee OA. The objective of this study was to examine knee OA over time to understand treatment and prevention strategies. Each site obtained approval from the respective institutional review board, and all participants signed a consent form before enrollment. For the current study, we used data from the baseline up to 96-month follow-up for participants with or at risk for knee OA.

### 2.1. Cohort Selection

Participants in the OAI aged 45 to 79 years were included and divided into three cohorts at the baseline: established knee OA cohort (n = 1389) for people who had symptomatic knee OA with the presence of both osteophytes and frequent knee symptoms in at least one knee; at risk of knee OA cohort (n = 3285) who had no symptomatic knee OA but were at risk for symptomatic OA in at least one knee; and a control cohort (n = 122 participants) with participants who had no symptomatic or radiographic knee OA and had no risk factors for knee OA. In this study, we used data for participants enrolled in the at risk of knee OA and established knee OA cohorts and followed from the baseline to the 96-month visit. The participants who were in the control cohort or had DM at baseline were excluded from the current study because healthy participants did not have symptoms that could affect gait speed and were at a lower risk of developing DM.

### 2.2. Outcome Measures

Diabetes was measured using self-reported Charlson Comorbidity Index (CCI) to identify DM incidence during follow-up visits by asking whether they had been diagnosed with DM (dichotomous). Prior research has reported the validity and reliability of using the CCI [36,37]. DM incidence was followed over the 4-time points at which the questionnaire was administered: 24 months, 48 months, 76 months, and 96 months follow-up.

### 2.3. Exposure Group

Gait speed was measured at baseline using a 20-m walk test. Participants were instructed to walk at their usual pace, wearing their usual footwear, and were permitted to use an assistive device if needed. The average of two trials was used to calculate the speed by dividing the distance (20 m) by the time required to complete the test [38] The gait speed was measured as meter/second (m/s).

### 2.4. Confounders

Body mass index (BMI) was measured as body mass (kg) divided by the square of height (m^2^). In this study, we used BMI values measured at baseline and follow-up because higher BMI has been strongly linked to DM. BMI was measured over 7-time points, including baseline, 12 months, 24 months, 36 months, 48 months, 72 months, and 96 months follow-up.

Age, gender (male or female), race, number of comorbidities, OA grade using Kellgren and Lawrence (KL) grade, and sub-cohort assignment (at risk or established knee OA) were included as covariates. Age was measured in years and categorized into four groups (i.e., <55 years, 56–65 years, 66–75 years, and >75 years). Race was categorized into four groups: White, African American, Asian, and Other. Number of comorbidities was measured using comorbidity score and categorized into four categories (i.e., none, one, two, three or more comorbidities). KL grade was obtained at baseline using x-ray with five categories ranging from 0 to 4 with greater score indicating worse knee OA. For this study, we included the worst KL grade for each participant as a covariate.

### 2.5. Statistical Analyses

Descriptive results were presented as means ± standard deviation (mean ± SD) for continuous variables or percentage for categorical variables. To compare gait speed at baseline between participants who developed incident DM and those who did not, independent t-tests were utilized. Incidence of DM between baseline and 96-month follow-up were examined as a cumulative incidence reported at 3-time points: 24 months, 48 months, and 96 months.

The incidence of DM was the primary outcome (yes/no). Generalized estimating equations (GEE) with a binary logistic regression analysis were used to evaluate the relationship between baseline gait speed and incidence of DM. GEE is a recommended longitudinal analysis when discrete time points are considered [39]. In addition, this analytical approach allows for including participants with missing data without excluding them from the model, allowing better estimation [40]. Therefore, GEE was conducted to allow for adjustments for confounders within subjects over time (i.e., BMI). Risk ratios (RR) with associated 95% confidence intervals (CI) were calculated. Three models were created: Model 1 (unadjusted) included only gait speed as a predictor and DM incidence as the primary outcome; model 2 was adjusted for age, sex, race, number of comorbidities, KL grades, and sub-cohort assignment; and model 3 was adjusted for age, sex, race, number of comorbidities, KL grades, sub-cohort assignment, and body mass index (at baseline and overtime).

We utilized a receiver operating characteristics (ROC) curve to determine the cutoff score for baseline gait speed that predicted incident DM. The area under the curve indicates the overall accuracy of the baseline gait speed in detecting the presence or absence of outcomes (e.g., DM incidence). Youden index was calculated (sensitivity + [1-specificity]) to determine the best cutoff score based on the largest Youden index. Finally, true positive and true negative were obtained using the number of true cases that are predicted as DM and the number of true non-cases that are predicted as no DM, respectively. True positive indicates sensitivity and true negative indicates specificity. The accuracy of the determined gait speed threshold was calculated using this formula: Accuracy = (sensitivity) (prevalence) + (specificity) (1—prevalence). Therefore, another GEE with a binary logistic regression was used to evaluate the relationship between the established cutoff score (slower vs. normal gait speed) and DM incidence after controlling for covariates. Three models were created similar to the previous models.

Sensitivity analyses were conducted after the removal of outliers for gait speed. Outliers were defined by gait speed greater than 3SD from the mean. Three models were created similar to the models for the total sample and the results were compared. All analyses were conducted by SPSS 25 for Mac. The alpha level was set at 0.05.

## 3. Results

**Figure 1:**
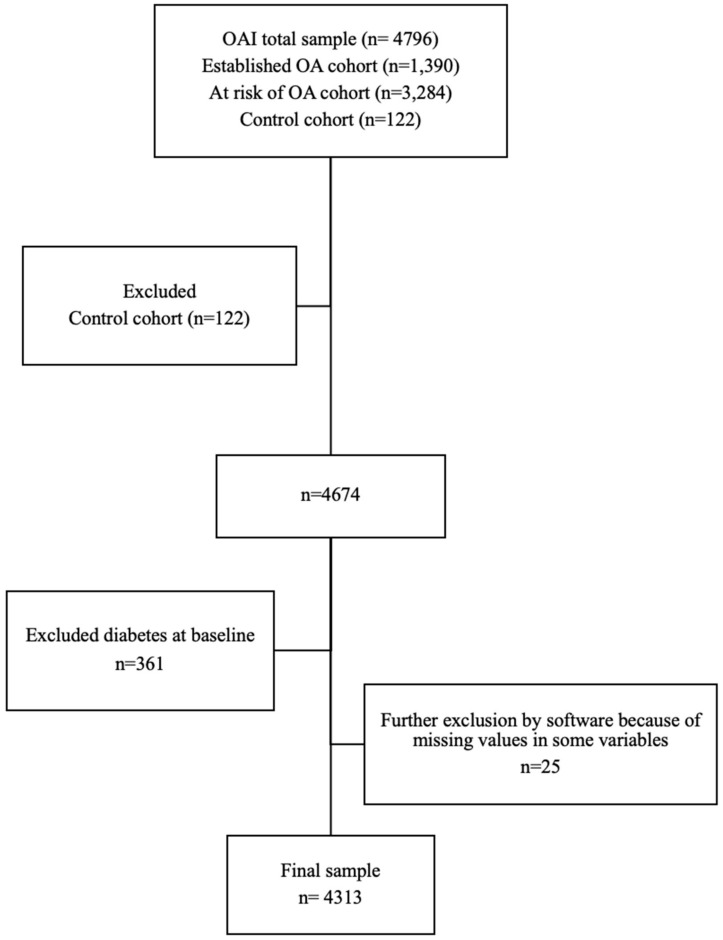
Flow chart of the participants’ selection.

A total of 4796 participants from the OAI were screened, and a total of 4313 were eligible for inclusion. Figure 1 shows the participants’ flow and the reasons for exclusion. Out of 4313 participants included in the analyses (mean age 61.1 ± 9.2, 58.7% females), 301 developed DM during a follow-up. The majority of participants (80.4%) were White. The mean ± SD of BMI at baseline was (28.5 ± 4.8 kg/m^2^) for the whole sample, reflecting an overweight/obese cohort. Table 1 shows the baseline characteristics for participants. The overall cumulative incidence of DM over eight years of follow-up was 7.0%, giving an overall DM incidence rate of 8.75 per 1000 person-years. Table 2 presents the cumulative incidence for DM at 3-time points (24-, 48- and 96-months follow-up).

Risk ratios for gait speed as a continuous variable predicting incident DM are presented in Table 3. Faster gait speed (as a continuous variable) was significantly associated with decreased risk of incident DM after adjustments for age, sex, race, number of comorbidities, KL grades, sub-cohort assignment, and body mass index (at baseline and overtime). Overall, participants with faster gait speed were about half as likely to develop incident DM over eight years of follow-up.

**Figure 2:**
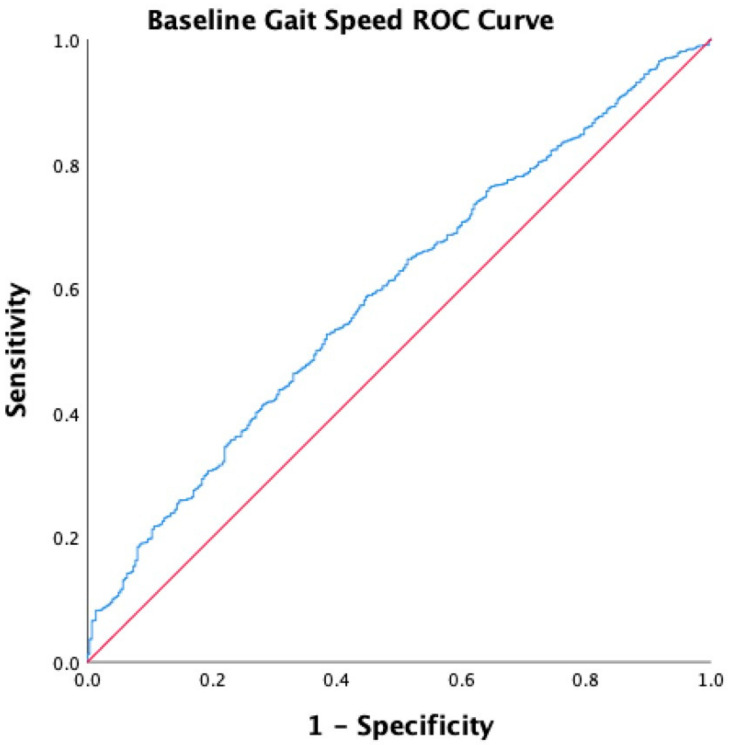
Receiver operating characteristic (ROC) curve for baseline gait speed.

We identified the cutoff value for baseline gait speed in predicting incident DM using a ROC curve. Our results found that the baseline gait speed predicted incident DM at a threshold of 1.32 m/s (sensitivity 0.53; specificity 0.62) with an area under the curve of 0.59 (95% CI [0.56–0.62], *p* < 0.001). The accuracy of this threshold for correct assignment of DM incidence based on gait speed was 0.61. Figure 2 shows the ROC results.

The analysis of the GEE with cutoff score is shown in Table 4 with the risk ratios for gait speed as a categorical variable predicting incident DM. Gait speed (<1.32 m/s) was a significant predictor for incident DM after adjustments for age, sex, race, number of comorbidities, KL grades, sub-cohort assignment, and body mass index (at baseline and overtime). Participants with slower gait speed were about 35% more likely to develop incident DM over eight years of follow-up.

The sensitivity analyses after exclusion of outliers were compared with the original analysis. A total of 24 cases were excluded due to extreme value of gait speed (m/s) distributed outside the mean ± 3SD. The results for GEEs were similar to the original analysis. In a fully adjusted model, higher gait speed (as a continuous variable) was significantly associated with decreased risk of incident DM (n = 3964, RR: 0.43; 95% CI [0.21–0.87], *p* = 0.044) after adjustments for age, sex, race, number of comorbidities, KL grade, sub-cohort assignment, and body mass index (at baseline and over time). The results from the ROC were similar to the original analysis. Baseline gait speed predicted incident DM at a threshold of 1.32 m/s (sensitivity 0.53; specificity 0.62) with an area under the curve of 0.59 (95% CI [0.55–0.62], *p* < 0.001). Finally, slower gait speed (<1.32 m/s) was a significant predictor for incident DM (n = 3964, RR: 1.34; 95% CI [1.01–1.79], *p* = 0.044) after adjustments for age, sex, race, number of comorbidities, KL grade, sub-cohort assignment, and body mass index (at baseline and overtime). Therefore, we chose to report the original analysis since there was no difference between outcomes before and after removing the outliers for baseline gait speed.

## 4. Discussion

The present study investigated the association between baseline gait speed and incident DM in adults with or elevated risk of knee OA. The results of this study indicate that higher gait speed was significantly associated with 56% lower risk for cumulative DM incidence in this cohort over 96 months after controlling for pertinent covariates. The results also showed that slower gait speed (<1.32 m/s) was a significant predictor for incident DM in this population. These results were consistent with a previous study that examined baseline gait speed and DM incidence in Japanese older adults over 4.16 years of follow-up, and found significant association [30]. However, the mean gait speed was higher in that study (1.48 m/s) than in our study (1.32 m/s). This discrepancy could be attributed to the small sample (n = 102) in Nakanishi’s study, shorter time of follow-up, and including only healthy older adults who participated in an exercise program for 9 years. Our study included individuals with or at risk of knee OA and identified a cutoff value for gait speed that predicted DM incidence in a larger cohort.

These findings concur with the results of a previous systematic review and meta-analysis of Louati and colleagues [22]. The results of that review included 49 studies revealed that the prevalence of DM was 14.4% in people with OA with an estimated ratio of 1.46 when compared to people without OA. The results of another recent systematic review and meta-analysis by Williams et al. (2018) [41] showed that people with musculoskeletal conditions such as neck, back pain, knee, or hip OA have a 17% increase in the rate of developing DM including cardiovascular disease and cancer. Our recent work with a total of 1255 participants reported that combined DM and arthritis was associated with reduced gait speed in general population [42]. This study identified the burden of those conditions on gait speed when compared to either condition (DM only and arthritis only) or no conditions. This indicates the importance of gait speed as a screening tool in the general population. Another population-based cohort study with a total of 16,362 participants aged 55 years and over showed a significant relationship between hip/knee OA and an elevated risk for incident DM of 16–25% after controlling for confounders [26]. In that study, baseline walking limitation explained 37–46% of the relationship between hip/knee OA and incident DM. However, these studies might differ from our study due to heterogeneity in definitions of OA and DM. Another difference is that these studies examined walking limitation as a self-reported outcome without measuring gait speed at baseline and without proper control for BMI over time.

The present study findings revealed that the risk of incident DM were 35% higher for participants with slower gait speed among people with or high risk of knee OA. These findings differ from those of some previous studies due to different research questions [43,44]. For example, a study has noted that DM is strongly associated with knee OA, but obesity may not be a confounding factor [43]. This study reported that the associations of type 1 DM and type 2 DM were 1.37 and 2.75 times higher, respectively, with knee OA among non-obese males and females aged 50–89 years compared to obese of both genders with the same age group. Another recent study concluded that using medications for DM was not associated with knee OA incidence, but independently reduced the progression of knee OA [44]. This study [44] had findings contrary to that of Eymard et al. (2015) [45] and Nieves-Plaza et al. (2013) [46], who found that DM was a risk factor for progression of knee OA after controlling for age, sex, BMI, hypertension, and dyslipidemia. A possible explanation for the results of those studies may be due to the concepts they used, different definitions for DM either as medications treated or self-reported diagnosis as an exposure and knee OA as an outcome. However, previous research has reported that medications such as antidiabetic, antilipemic, and antihypertensives might be associated with decreased incidence or progression of OA [47,48,49] or affect pain [35]. Another possible explanation for this is that the lack of gait speed role in the association of knee OA and DM incidence and the definitions of DM and OA used in those studies. Our previous report found that DM was associated with decreased gait speed in people with knee OA [29].

Sensitivity and specificity results for the threshold of gait speed are not very high. The area under the curve in the current study (0.59) was below the acceptable threshold (0.7) [50]. This value indicates a 59% chance that health care providers will correctly distinguish patients who are at risk of DM incidence based on gait speed. The cutoff value of gait speed of 1.32 with sensitivity 0.53 and specificity 0.62 was significantly associated with DM incidence. Clinicians may consider this cutoff (1.32) as a screening value for DM incidence, but this value may not be considered a slow gait speed. However, this value was higher than previously determined gait speed cutoff (<1.2 m/s), which is associated with difficulty in crossing streets or community ambulation [51,52], functional limitations [53], and mortality [10]. Therefore, findings and cutoff values of the gait speed in the current study may require confirmation in future studies.

The current study findings may be somewhat limited by not including potential confounders such as family history of DM, duration of DM, and medications. The results should be interpreted with caution because knee OA severity over time was not assessed, in that we adjusted KL grade at the baseline only. Moreover, DM was self-reported, relying on the individuals’ recall, which could introduce misclassification bias. However, the validity and reliability of using self-reported diabetes have been reported previously [36,37]. Another limitation of this study was that type 1 or type 2 DM was not distinguished by using the CCI. The study strength includes a reliance on a large population cohort study, the valid objectively measured gait speed [38]. Thus, we believe that the current study findings are generalizable to people with or at elevated risk for knee OA.

The results of the current study provide concrete goals that could inform the clinicians’ approach to interpreting gait speed, an essential marker of health [12], especially in people with or at high risk of knee OA. Weight loss and improving physical functions and activity level might be considered targets for treatment as they have influence on gait speed. Previous studies have reported that people with symptomatic knee OA had an almost 9-fold risk of fast gait speed decline compared to those with neither pain nor radiographic knee OA [54]. The current study results suggest to policymakers, researchers, and clinicians that gait speed is a clinically relevant measure and could possibly be used as a prognostic factor for DM incidence in people with or at elevated risk for knee OA. These findings have important implications for clinicians in identifying those at risk for developing DM through using gait speed as a marker for elevated risk. Our research has established a cutoff score of gait speed that predicts incident DM in people with or at elevated risk for knee OA. Future research should examine this cutoff (<1.32 m/s) in different samples and populations.

## 5. Conclusions

The present study was designed to examine the utility of baseline gait speed in predicting diabetes incidence among people with or at elevated risk for knee OA. The results show the value of baseline gait speed in predicting incident diabetes in this population, even after controlling for BMI at baseline and over time, along with other covariates. Furthermore, a cutoff for baseline gait speed was established at a threshold of 1.32 m/s, which was significantly associated with DM incidence. Therefore, slower baseline gait speed significantly predicted development of DM over 96 months of follow-up. The findings advance understanding regarding the predictive ability of gait speed for the development of incident DM in this population. Further research needs to examine the links between gait speed and DM biomarkers such as serum glucose level.

## Figures and Tables

**Table 1:** Baseline characteristics.

Characteristics	Total Sample (N = 4313)
Age categories	
<55 years, n (%)	1395 (32.3)
56–65 years, n (%)	1413 (32.8)
66–75 years, n (%)	1213 (28.1)
>76 years, n (%)	292 (6.8)
Sex, female, n (%)	2532 (58.7)
Race	
White, n (%)	3469 (80.4)
African American, n (%)	735 (17.0)
Asian, n (%)	34 (0.8)
Others, n (%)	70 (1.6)
Missing, n (%)	5 (0.1)
Number of comorbidities	
None, n (%)	3404 (78.9)
One, n (%)	543 (12.6)
Two, n (%)	227 (5.3)
Three or more, n (%)	77 (1.8)
Missing, n (%)	62 (1.4)
Baseline Body Mass Index, mean (SD)	28.5 (4.8)
Kellgren and Lawrence grade	
Grade 0, n (%)	1110 (25.7)
Grade 1, n (%)	624 (14.5)
Grade 2, n (%)	1249 (29.0)
Grade 3, n (%)	808 (18.7)
Grade 4, n (%)	266 (6.2)
Missing, n (%)	256 (5.9)
Sub-cohort assignment	
At risk of knee OA cohort, n (%)	3074 (71.3)
Established knee OA cohort, n (%)	1239 (28.7)
Gait speed, m/s, mean (SD)	1.32 (0.21)

**Table 2:** Cumulative incidence of DM.

Cumulative Incidence Time	Study Sample N = 4288 Participants with Incident DM, N (%)
At 24 months	95 (2.2)
At 48 months	181 (4.2)
At 96 months	301 (7.0)

**Table 3:** Risk ratio for the incidence of DM by gait speed as a continuous variable.

	N	RR [95% CI]	*p*-Value
Model 1	4274	0.21 [0.13, 0.35]	<0.001
Model 2	3983	0.24 [0.13, 0.44]	<0.001
Model 3	3983	0.44 [0.22, 0.86]	0.018

Number of participants with incident DM = 301 (7%) of the total participants. RR: Risk ratio, CI: confidence interval; Model 1: unadjusted; Model 2: adjusted for age, sex, race, number of comorbidities, KL grades, and sub-cohort assignment; Model 3: adjusted for age, sex, race, number of comorbidities, KL grades, sub-cohort assignment, and body mass index (at baseline and overtime).

**Table 4:** Risk ratio for the incidence of DM by gait speed as a categorical variable with a cutoff of 1.32 m/s.

	N	RR [95% CI]	*p*-Value
Model 1	4274	1.77 [1.39, 2.26]	<0.001
Model 2	3983	1.70 [1.29, 2.23]	<0.001
Model 3	3983	1.35 [1.01, 1.79]	0.042

RR: risk ratio, CI: confidence interval; Model 1: unadjusted; Model 2: adjusted for age, sex, race, number of comorbidities, KL grades, and sub-cohort assignment; Model 3: adjusted for age, sex, race, number of comorbidities, KL grades, sub-cohort assignment, and body mass index (at baseline and overtime).

## Data Availability

Data are available via https://data-archive.nimh.nih.gov/oai/ (accessed on 20 April 2021).
